# 

**Published:** 1884-02

**Authors:** 


### EDITORIAL.

### Mississippi Valley Dental Society.

The Fortieth Annual Meeting of this Pioneer Society will be held in the lecture room of the Ohio College of Dental Surgery, on Wednesday, March 5th, at 10 o'clock A. M.

It is desirable that there be a large attendance. On two or three accounts this society should receive the respect, support, and co-operation of the dental profession in this region of the country. Its age, to say the least, entitles it to respect. Its course has been one of uniform interest and success. The results that have been accomplished by it cannot be estimated. Its influence has always been on the progressive side of all important professional questions, and it has done much in giving direction to thought and practice, in many particulars. It was in operation many years before it could have any aid or support from similar associations. It had to mark out its own course, and do its own work; and when its whole course is reviewed it is remarkable how few mistakes have been made in its work. Those who organized this society have nearly all passed away, but the spirit and energy which they exhibited have been, to some extent at least, entailed to their successors.

They have left a noble example, one worthy of all imitation. Such names as M. Rogers, Jesse W. Cook, B. B. Brown, Joseph Sanford, W. E. Ide, S. P. Hullihen, Edward Taylor, Jas. Taylor, Joseph Taylor, Sam'l Griffith, W. H. Goddard, D. P. Hunt should ever be held in grateful remembrance, especially by all those who were witnesses of their devoted labors, and all, who in the future may be members, as they study the history of this society, will not fail to render the honor due to the men who laid so deep and broad foundations for those who should come after them.

The approaching meeting will be one of much interest, and it is to be hoped will draw a large attendance, and that all will come with the resolution and preparation, to make the occasion one of interest and profit.

To further this end so far as they can, the Executive Committee have presented the following programme:

1. Artificial crowns upon roots; best method of adapting them.

2. What means are best adapted for maintaining the teeth and mouth in a healthy condition?

3. What progress has been made in arresting and preventing decay of the teeth, in the last five years?

4. In what respect, if any, are decayed teeth better filled now than ten years ago?

5. What valuable medicinal agents have been introduced into dental practice in the last three years? For what purpose is each one used?

6. What improvements, if any, have been made in artificial dentures, during the last five years ; and what further improvements are desirable?

These subjects are somewhat out of the usual line, but they give a wide range for thought and investigation. Let every member improve it. The REGISTER will have a reporter and a full report of the proceedings.

### Commencement.

The Annual Commencement of the Ohio College of Dental Surgery will be held in College Hall, Walnut Street, on Tuesday evening, March 4th, at 8 o'clock. All interested are cordially invited.

### College Association.

The annual meeting of the Ohio Dental College Association will be held in the auditorium of the College on Tuesday, March 4th, at 2 o'clock P. M. A full attendance of the members is very desirable.

### THE DENTAL REGISTER,

A MONTHLY MAGAZINE DEVOTED TO THE INTERESTS OF THE

### DENTAL PROFESSION.

*Terms Reduced to* $2.00 *Per Year. Single Copies,* 25 *Cents.*

**EDITED BY PROF. J. TAFT, D.D.S.**

AND

**Published by SPENCER & CROCKER, Ohio Dental and Surgical Depot.**

The volume commences in January and closes in December.

The REGISTER being issued monthly is always fresh, therefore Indispensable to the practitioner who desires to keep posted up with the new inventions and improvements which are daily developed. Neither expense nor effort will be spared to give value and variety to its contents Articles will be illustrated with well executed engravings whenever they will add to the interest or assist to explanation.

Its extensive circulation and frequency of issue make it one of the BEST DENTAL ADVERTISING MEDIUMS in the United States.

All subscriptions must begin with January or July.

Local or special notices, five lines or less, will be inserted for $3 each insertion; over five lines, 50 cts. per line.

All advertising bills payable in advance, or at furthest, after the issue of the first number containing the advertisement. All transient advertisements to be paid for in advance.

☞CONTRIBUTIONS SOLICITED.

Exclusively Editorial Communications should be addressed to the Editor.

The latest number of the REGISTER may be ordered with or without other goods at any time, and all letters should be addressed to

**SPENCER & CROCKER, Ohio Dental & Surgical Depot, Cincinnati, O.**

